# Clinical and Biomechanical Outcomes of One-Stage Treatment of a Simultaneous Ipsilateral Patellar Tendon and ACL Tear Combined with a Tibial Plateau Fracture: A Case Study

**DOI:** 10.1155/2020/5793948

**Published:** 2020-02-04

**Authors:** Petros Ismailidis, Rolf Kernen, Christian Egloff, Corina Nüesch, Annegret Mündermann, Sebastian Andreas Müller

**Affiliations:** ^1^Department of Orthopaedics and Traumatology, University Hospital Basel, Spitalstrasse 21, 4031 Basel, Switzerland; ^2^Department of Biomedical Engineering, University of Basel, Gewerbestrasse 14, 4123 Allschwil, Switzerland; ^3^Department of Clinical Research, University of Basel, Schanzenstrasse 55, 4056 Basel, Switzerland; ^4^Clinic for Orthopedics “Claraortho”, Claragraben 82 4058 Basel, Switzerland

## Abstract

Simultaneous ipsilateral patellar tendon (PT) and anterior cruciate ligament (ACL) tear is a rare injury. Associated meniscal and ligamentous injuries are common but frequently initially missed. In contrast, to date, there is no report of associated fractures. We report on a 40-year-old female Caucasian patient presenting with a ski injury resulting in simultaneous ipsilateral patellar tendon and ACL tear combined with a tibia plateau fracture and a medial and lateral meniscus lesion. ORIF of the tibia as well as one-stage primary reconstruction of the PT and ACL and suturing of the menisci was conducted. The final follow-up was 2 years postoperatively. Lower extremity kinematic, kinetic, and muscle activity measurements were conducted. Although the clinical result was excellent, altered joint kinematics went along with large side-to-side difference in hip and knee joint moments during midstance and terminal stance. During weight acceptance, vastus medialis and hamstring muscles showed greater relative activity in the injured than the uninjured side. This case demonstrates the possibility of excellent early and midterm results with a one-stage approach and suitable rehabilitation scheme. Biomechanical measurements could further help evaluate the outcome of the treatments and implications for the development of potential secondary damage.

## 1. Introduction

Simultaneous ipsilateral patellar tendon (PT) and anterior cruciate ligament (ACL) tear is a rare injury. A systematic review in 2018 identified 28 cases in 17 published reports in the English literature [1]. Associated meniscal and ligamentous injuries are common but frequently initially missed [1]. In contrast, to date, there is no report of associated fractures. Moreover, there are no established treatment algorithms: open and arthroscopic approaches, as well as one- or two-stage approaches, have been described [2]. In our study, we report on a patellar tendon and ACL tear combined with a tibia plateau fracture in a middle age physically active female patient.

## 2. Case Report

**Figure 1:**
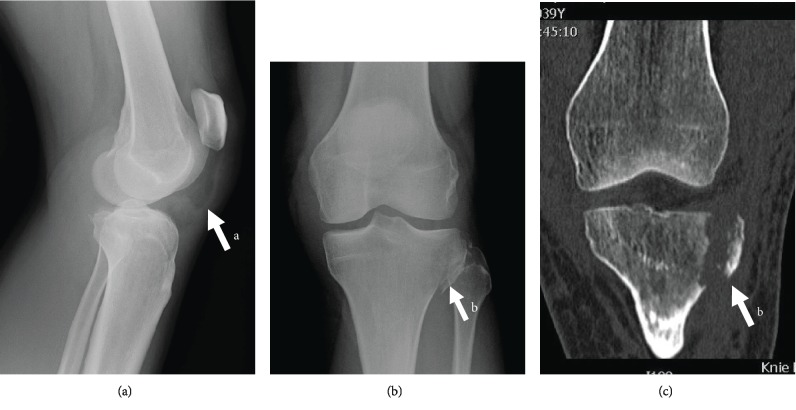
Preoperative X-rays: anterior-posterior (a), lateral (b), and coronal (c) view CT. Note the patella alta with a Caton Deschamps index of 1.5 (A) as an indirect sign of the PT rupture, as well as tibial plateau fracture (B).

Written patient consent was received and archived. A 40-year-old patient without any preexisting injury or morbidity suffered a knee injury while skiing. The exact injury mechanism was not clear; however, the patient described no direct impact and recalls a twisting injury. The patient initially presented herself to a local health center. The X-rays revealed a fracture of the tibia. Both the PT and the ACL rupture were initially overlooked. The patient was referred to our institution for the definitive treatment. On presentation at our institution, there was a large knee effusion with a palpable infrapatellar gap. Straight leg raise was impossible. X-ray analysis (Figure 1) revealed—in addition to the lateral tibial plateau fracture—a patella alta (Caton Deschamps index [3] of 1.5) and raised the suspicion of a PT injury. Specific ACL tests were not reliable because of the large effusion and pain, which did not allow manipulation required for the tests. The CT scan (Figure 1) allowed the further evaluation of the tibial plateau fracture (Schatzker type 1 [4]) and the proximal migration of the patella and confirmed the diagnosis of PT rupture. The patient was scheduled for surgery on the following day with the diagnosis of a tibial plateau fracture and PT rupture. No MRI was conducted.

**Figure 2:**
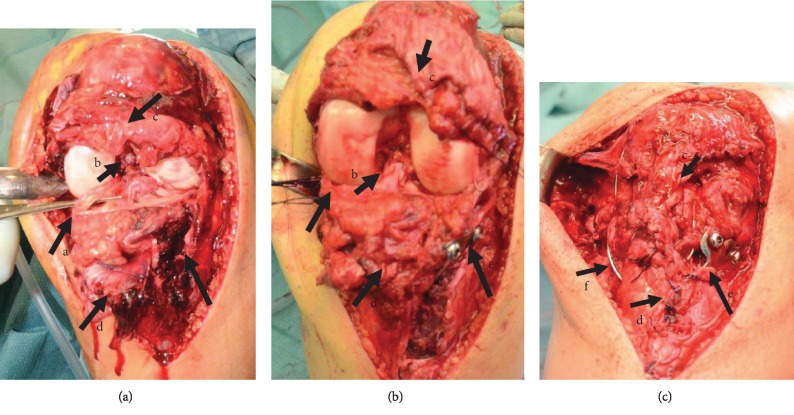
Intraoperative photos after the exposure of the joint (a); after the ORIF of the tibia, ACL reconstruction, and meniscus suturing (b); and after the PT repair (c). Note on the figures: (A) bucket handle tear of the medial meniscus before and after suturing, (B) absence of the ACL in the notch, the tibial stump of the ACL, and the reconstructed ACL, (C) the proximal and (D) distal stump of the PT before and after reconstruction, (E) the tibial plateau fracture before and after ORIF, and (F) the McLaughlin wire.

The operation was performed through an anterior midline incision. The diagnosis of a full thickness PT tear was confirmed, and a complete proximal tear of the ACL as well as a bucket handle injury of both menisci was diagnosed (Figure 2(a)). We decided to immediately suture both menisci and to perform a primary repair of the ACL and PT. Furthermore, there were chondral lesion Grades I-II [5] at both femoral condyles that were not addressed surgically.

**Figure 3:**
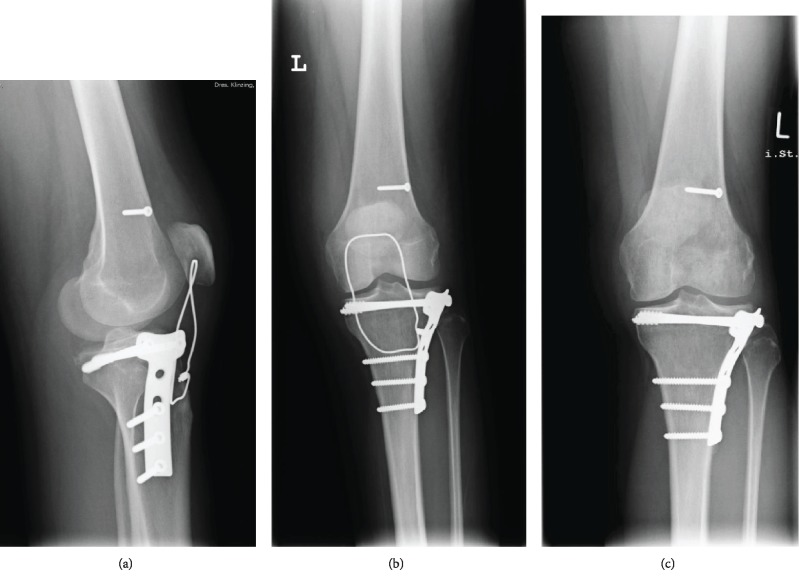
Radiographic result after the operation (a, b) and after the removal of the McLaughlin wire (c).

The tibial plateau fracture was reduced and fixed with a lateral 4.5 mm T-Plate (Figures 2 and 3). The plate was manually contoured to best fit the tibia anatomy, the proximal screws were partially threaded to achieve interfragmentary compression, and the rest of the screws were normal cortex screws. The ACL was ruptured on the femoral side (Sherman type 1 [6]). The tibial stump was anchored 5 mm Mersilene Tape (Ethicon, Somerville, NJ) whip stitches and then passed through a femoral tunnel at the femoral origin of the ACL and secured on the femoral side with a 3.5 mm screw used as a suture post. The menisci were sutured with horizontal inside-out mattress stitches using number 2-0 Mersilene (Ethicon, Somerville, NJ) sutures. Finally, the PT was repaired using number 2 Vicryl whip stitches and secured with a McLaughlin cerclage with a 1.5 mm wire (Figures 2 and 3).

The knee was protected in a mobile brace for 6 weeks. The patient was mobilized with protected weight bearing of 15 kg for 6 weeks; the allowed range of motion (ROM) was restricted to 30° of flexion and gradually increased in increments of 30° every 2 weeks. The early physiotherapy focused on gradually regaining ROM including contiguous passive motion and quadriceps function with closed chain exercises. Stationary cycling and proprioceptive exercises were initiated 4 months posttrauma. Running was initiated at 6 months.

**Figure 4:**
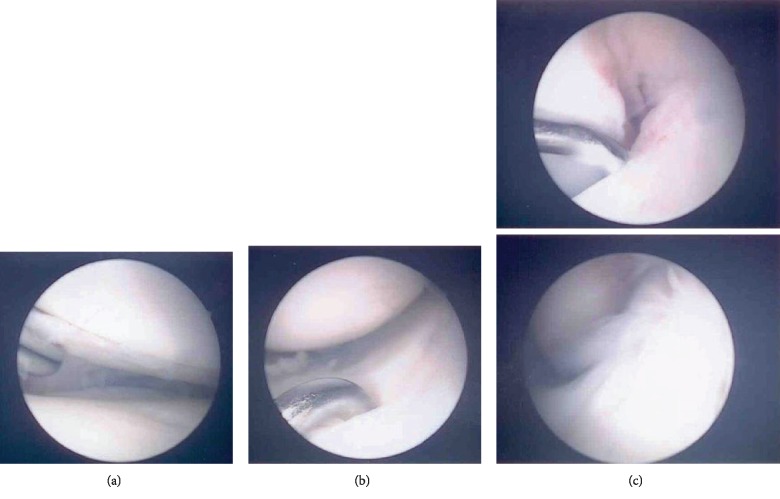
Arthroscopic views of the medial (a) and lateral (b) meniscus and the ACL (c) 3 months postoperatively. The ACL and both menisci were healed.

The postoperative follow-up showed no complications. The patient had a ROM of 90-0-0° 6 weeks after surgery. There was no breakage of the cerclage. Three months postoperatively, the cerclage was removed and a diagnostic arthroscopy was performed. The arthroscopy showed complete healing of the ACL and both menisci (Figure 4). The return to daily activities was uncomplicated already 6 months postoperatively. One year postoperatively, the lateral tibia plate was removed. A control arthroscopy was recommended. This would allow us to inspect the menisci and ACL reconstruction as well as perform a notchplasty in case of an intraoperatively ascertained ACL impingement. The arthroscopy was not performed because of the lack of cost coverage by the health insurance company and additionally because the patient was fully satisfied with the result and wanted to limit the intervention to the implant removal only.

The final follow-up was 2 years postoperatively: the IKDC Score was 90, the Lysholm Score 94, and the ROM 145-5-0° (contralateral ROM 150-0-10°). Isokinetic knee flexor and extensor strength tests were performed at 60°/s and 240°/s using an isokinetic dynamometer (Biodex System 4 Pro: Biodex Medical Systems, Shirley, NY, USA). At 240°/s, knee flexor strength was 5.0% higher and knee extensor strength and work were 20% and 22% lower, respectively, in the injured leg than in the contralateral leg. At 60°/s, knee flexor strength was 5% higher and knee extensor strength and work were 7% and 8% lower, respectively, with a clear delay in force production of the knee extensors in the injured leg compared to the contralateral leg.

**Figure 5:**
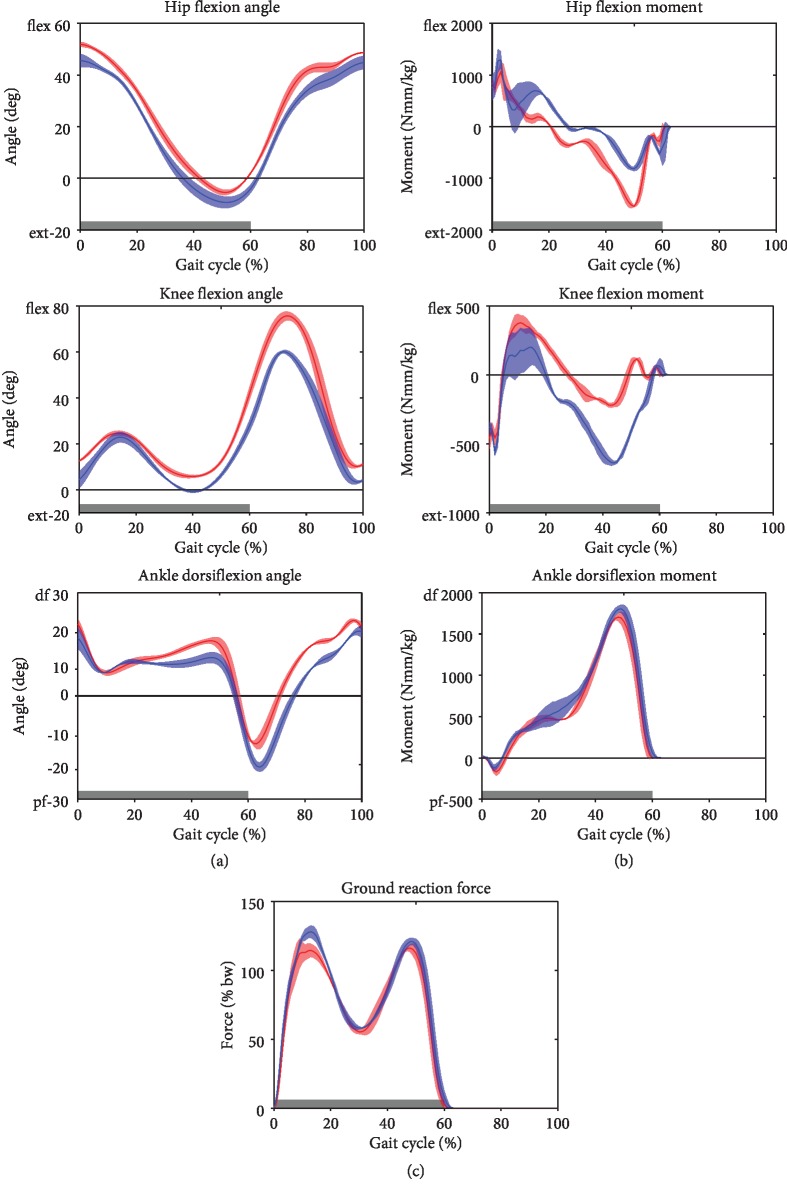
Joint angles (a) and resultant external joint moments (b) in the sagittal plane and vertical ground reaction force (c) during walking. Gait data was normalized to one gait cycle.

**Figure 6:**
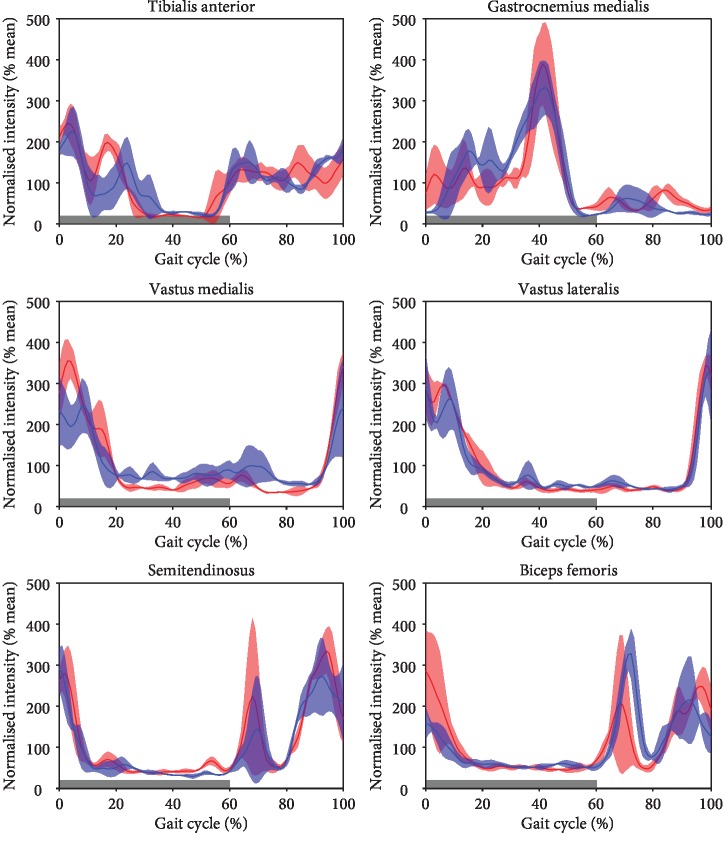
Relative muscle activity of selected lower extremity muscles during walking. Gait data was normalized to one gait cycle.

Lower extremity kinematic and kinetic data and muscle activity were collected during overground walking at 1.7 ± 0.1 m/s using an 8-camera motion analysis system (Vicon, Oxford, UK; sampling rate: 120 Hz), two force plates (Kistler, Winterthur, Switzerland; sampling rate: 2400 Hz), and a 12-channel surface electromyography (EMG) system (myon AG, Schwarzenberg, Switzerland, sampling rate 2400 Hz). Throughout the gait cycle, both the hip and the knee were in more flexed positions on the injured than the uninjured side (Figure 5). The injured knee presented a 6° extension deficit during terminal stance and a 16° higher flexion throughout swing. This altered joint kinematics went along with large side-to-side difference in hip and knee joint moments during midstance and terminal stance (Figure 5). During this interval, knee extension moments were 65% lower and hip extension moments were 89% higher in the injured than the uninjured side despite similar vertical ground reaction forces in both sides. From terminal stance through swing, the ankle on the injured side was approximately 6° more dorsiflexed than that on the uninjured side. In midstance, the gastrocnemius medialis muscle was less activated and the ankle dorsiflexion moment was 6% smaller on the injured side than the uninjured side (Figure 6). The tibialis anterior and vastus lateralis muscles showed similar activities on both sides throughout the gait cycle. During weight acceptance, vastus medialis and hamstring muscles showed greater relative activity in the injured than the uninjured side. During early swing, relative hamstring muscle activity was lower in the injured than uninjured side.

## 3. Discussion

Simultaneous ipsilateral PT and ACL tear is a rare injury with only very few studies reporting on this injury, the largest of which reports on 6 patients [7]. Hence, there is no clear evidence on the optimal diagnostic procedures, treatment, or expected outcomes. However, the existing literature reported a few interesting findings.

### 3.1. Associated Injuries

With the exception of two reported cases [7, 8], combined PT and ACL tears always occurred with associated ligamentous or meniscal injuries. While only one case reported a knee dislocation on the initial X-rays [9], a dislocation and spontaneous reduction before the consultation could be possible in our case as in all other cases reporting on PT and ACL injury. Rupture of the medial collateral ligament (MCL) was the most frequent (66.0%) ligament injury, and 69.0% of patients had an injury in either one or both menisci [1]. In contrast, to date, there is no report of associated fractures. PT and ACL rupture or the associated injuries are commonly missed at initial clinical and radiological diagnostics and only diagnosed intraoperatively. Cumulatively, in 43.3% of the reported cases, the ACL or the PT rupture was initially missed [1]. While in these studies, X-rays of the knee in two planes were the standard diagnostic procedure; preoperative CT or MRI was not conducted routinely. In our case, a part of the injury was also missed by the initial diagnostic procedures.

### 3.2. Treatment Strategies

Previously reported cases were treated with various treatment strategies. Apart from one case where the patient was presented with a delay of 2 months [8], the PT injury was always directly repaired, while in most reports (75%), the ACL was also addressed surgically. Of these, 50% were one-stage procedures (primary PT repair and ACL reconstruction), and the other 50% were two-stage procedures (primary PT repair followed by ACL reconstruction) [1]. The rehabilitation scheme differed between reported cases: most authors prescribed some kind of ROM restriction similar to that commonly used after PT reconstructions and in contrast to the early ROM mobilization usually used after ACL reconstruction. The rehabilitation of the PT repair (restricted ROM) theoretically conflicts with that of the ACL (early ROM encouraged) leading some authors to the decision of waiting for the full rehabilitation of the PT injury before addressing the ACL. In our opinion and as demonstrated in this case, a combined scheme is feasible; the rehabilitation alone should be no reason to avoid a one-stage approach, especially if the ACL is avulsed proximally and amenable for a primary repair. Although no specific literature for combined ACL and PT injuries exists, current concepts of treating multiligament injuries in the knee also imply this principle: all injured structures should be reconstructed concurrently [10] so that an early postoperative knee ROM can be initiated in order to minimize the risk of postoperative stiffness.

Of the authors reporting an ACL reconstruction, 77% used an autograft while the rest used an allograft or a prosthetic ligament [7]. None of the surgeons reported on a direct reconstruction of the ACL. Some authors of previous reports preferred an arthroscopic ACL reconstruction and reported good results using this technique [7]. Arthroscopic ACL reconstruction with autograft or allograft is a standardized procedure with usually good results [11]. However, we believe that—because an open approach is necessary for the PT reconstruction—the ACL repair can be conducted directly through the same approach. In contrast to an autograft or allograft reconstruction, a direct repair seems like a risky option. Historically, direct repair of the ACL has been widely abandoned in favor of ACL reconstruction because of the unpredictable results and the high rates of failure. However, recently, interest on direct repair has increased again with animal and human studies providing promising results given careful patient selection [12]. In the presence of a proximal tear, a direct repair appears to be a good option sparing the need for an autograft and offering the advantages of preserving proprioception and better reconstructing knee kinematics above all with the addition of an internal reinforcing bracing device [12].

### 3.3. Outcome

Good outcomes were reported for one- and two-stage approaches and for all surgical methods including arthroscopic and open arthroscopic-assisted techniques as well as open surgery. In all but one reported cases [8], patients achieved full knee extension and knee flexion above 120°. The follow-up varied from 6 months to 4 years. However, specific orthopedic scores were documented in only less than half of the cases, and strength measurements were only performed in three studies [13–15]. Although return to daily activities and sport is expected after a surgical repair, most studies did not specify the time frame in which this was achieved [2]. Although postoperative complications such as patella baja, surgical site infection [7], complex regional pain syndrome and stiffness [8], persistent patellofemoral crepitus [16], and arthrofibrosis [17] were reported, these complications did not negatively influence the long-term result except in one case [8] in which the partial PT rupture was not addressed surgically. In two systematic reviews on this topic, no significant difference in the outcome between the one- and two-stage approaches was detected [1, 2]. However, one of these reviews reported a significantly higher complication rate in the one-stage approach [2].

### 3.4. Gait and Functional Analysis

None of the previous studies has reported on gait analysis or EMG measurements. In our case, the clinically assessed extension deficit of 5° was also portrayed in the kinematics. Hence, the extension of the injured side was reduced through the stance phase, resulting in a higher knee flexion moment (Figure 5). Through the lack of full knee extension, the activity of the vastus medialis and biceps femoris necessary to stabilize the leg through the initial foot contact of the stance phase was increased, resulting in increased relative muscle intensity (Figure 6). Less knee extension during the terminal stance was associated with lower knee extension moments during the terminal stance in the injured limb and higher than normal knee extension moments in the uninjured limb indicating an overload of the contralateral knee. This underlines the importance of increased ROM and particularly of achieving full extension by anatomical ACL reconstruction avoiding anterior graft impingement and by early rehabilitation. Moreover, these results illustrate the need of kinematic and kinetic analysis to fully understand the functional results of the injury and prevent long-term secondary changes not only in the affected limb but also in the contralateral limb. In our case, the extension deficit of only 5°—presumably caused by the injury and clinically judged as not important—appears to have a significant impact on the gait biomechanics of the patient.

Only three previous studies on ACL and PT rupture performed strength measurements [13–15]. For instance, Mariani et al. [14] reported on three cases evaluating isometric strength in extension at 90° knee flexion reporting a deficit compared to the contralateral limb of 21, 24, and 6%, respectively. Cucchi et al. [13] measured isometric strength in knee extension from a starting position with the knee and hip flexed 90° in two patients and reported a strength deficit compared to the contralateral limb of −15% and +20%, respectively. Pérez et al. [15] performed measurements using a manual manometer and reported identical power of quadriceps and hamstrings of the injured leg compared to the contralateral side on the last follow-up of 18 months postoperatively. In our study, we performed isokinetic measurements and measured an increase of 5% in maximum knee flexor strength and a reduction of 19.6% in maximum knee extensor strength. Clearly, gradual strengthening of the extensor muscles is an important goal of the rehabilitation. While the real impact of the injury on the reduction in muscle strength and the period needed to recover is difficult to assess from these individual reports, there was still a knee extensor strength deficit at the final follow-up in 5 of the 7 patients measured [13–15]. This observation is concurrent in our patient who had lower knee extensor strength in the injured side. However, a more flexed knee position requires greater muscle forces represented as greater knee flexion moments during the first half of the stance. The lower force production capacity of the knee extensor muscle in the injured leg explains the greater relative muscle activity of the extensor muscles observed in the first half of the stance. Strengthening knee extensors in the injured limb may not only normalize joint motion during gait but also shift loads (measured by the surrogate joint moment) towards normal values and hence positively affect the long-term outcome after this complex injury.

## 4. Conclusion

To the best of our knowledge, this is the first case reporting on a simultaneous ACL and PT injury combined with a tibial plateau fracture in a nondislocated knee.

Overall, this case adds to the existing knowledge regarding the treatment of simultaneous PT and ACL rupture and emphasizes the need for extensive diagnostic procedures to capture and diagnose possible concomitant injuries. Moreover, the surgeon should rule out additional injuries intraoperatively. This case further shows the potential of excellent early and midterm functional results with a one-stage approach and suitable rehabilitation scheme. Lastly, our results confirm the direct repair as a possible operative technique in cases with proximal rupture of the ACL. Clearly, there is a need for further reports to fully understand and optimally treat combined ACL and PT injuries. Biomechanical measurements could further help evaluate the outcome of the treatments and implications for the development of potential secondary damage.

## Abbreviations

ACL: Anterior cruciate ligament
PT: Patellar tendon
ORIF: Open reduction internal fixation.
